# Supplementary material for: Cancer cell plasticity defines response to immunotherapy in cutaneous squamous cell carcinoma
Source: Nat Commun. 2024 Jun 24;15:5352. doi: 10.1038/s41467-024-49718-8 (PMC11196727; doi:10.1038/s41467-024-49718-8)
Supplement: Supplementary file 1 — Supplementary Information [file 41467_2024_49718_MOESM1_ESM.pdf]

Supplementary Fig. 1

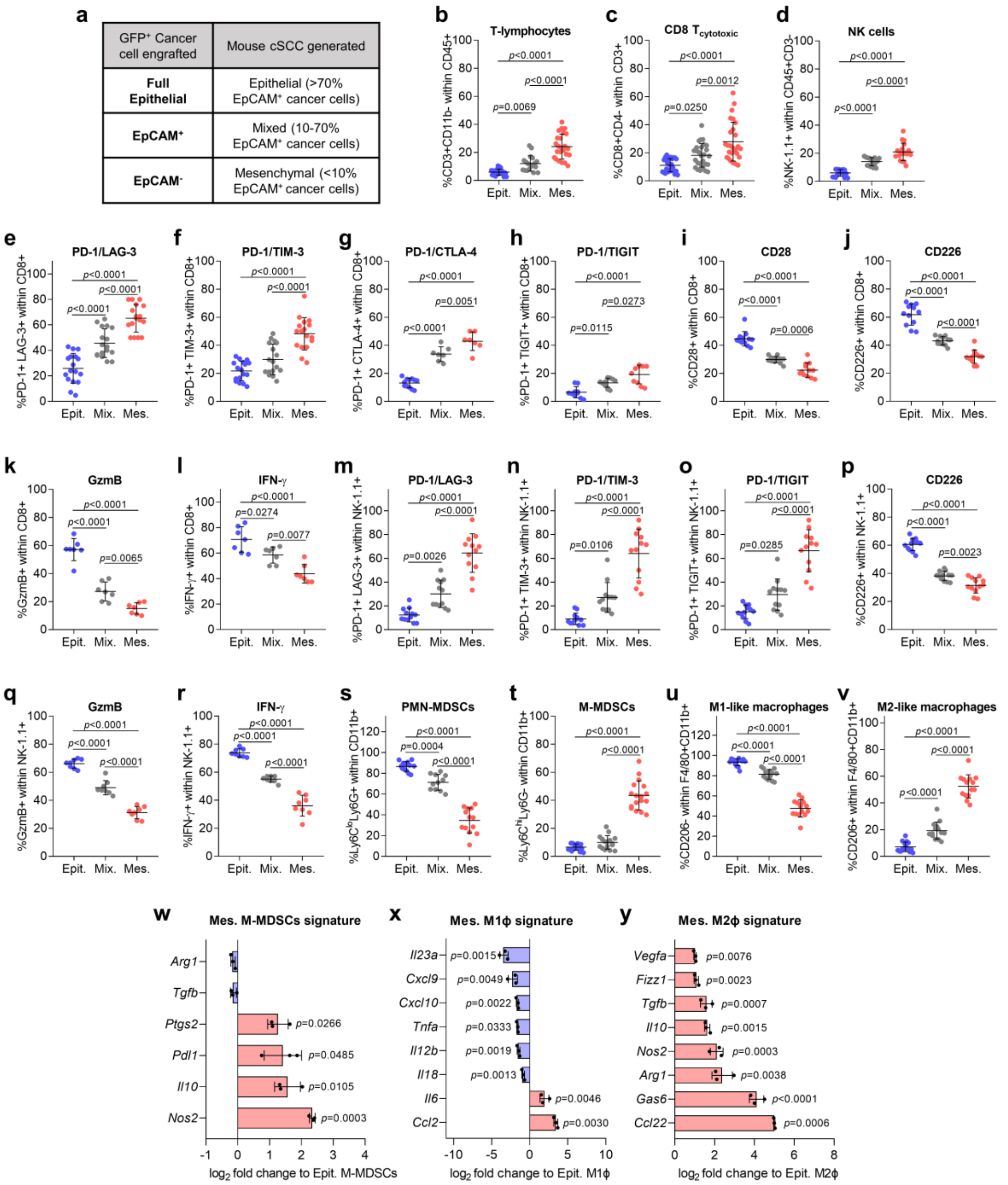

Supplementary Figure 1. Exhausted CTLs and NK cells, and immunosuppressive immune cells increase during mouse cSCC progression. a, Epithelial, mixed and mesenchymal cSCCs were generated after engrafting full epithelial cancer cells from WD-SCCs, and EpCAM$^+$ and EpCAM$^-$ cancer cells from MD/PD-SCCs, respectively, into immunocompetent syngeneic mice. **b-d**, Percentage of (**b**) T lymphocytes (CD3$^+$CD11b$^-$ within CD45$^+$; n = 24 per group), (**c**) CD8$^+$ T cells (CD8$^+$CD4$^-$ within CD3$^+$; n = 27 per group) and (**d**) NK cells (NK-1.1$^+$ within CD45$^+$CD3$^-$; n = 18 per group) in the indicated mouse cSCCs. **e-l**, Percentage of (**e**) PD-1$^+$LAG-3$^+$ (n = 17 per group), (**f**) PD-1$^+$TIM-3$^+$ (n = 17 per group), (**g**) PD-1$^+$CTLA-4$^+$ (Epit.: n = 12; Mix./Mes: n = 7), (**h**) PD-1$^+$TIGIT$^+$ (n = 10 per group), (**i**) CD28$^+$ (n = 12 per group), (**j**) CD226$^+$ (n = 12 per

group), (**k**) GzmB$^+$ (n = 7 per group) and (**l**) IFN-γ^+ (n = 7 per group) cells within CD8$^+$ T cell population in the indicated mouse cSCCs. **m-r**, Percentage of (**m**) PD-1$^+$LAG-3$^+$ (n = 12 per group), (**n**) PD-1$^+$TIM-3$^+$ (n = 12 per group), (**o**) PD-1$^+$TIGIT$^+$ (n = 12 per group), (**p**) CD226$^+$ (n = 12 per group), (**q**) GzmB$^+$ (n = 8 per group) and (**r**) IFN-γ^+ (n = 8 per group) cells within NK cell population in the indicated mouse cSCCs. **s-v**, Percentage of (**s**) PMN-MDSCs (Ly6C^{lo}Ly6G$^+$ within CD11b$^+$; n = 13 per group), (**t**) M-MDSCs (Ly6C^{hi}Ly6G$^-$ within CD11b$^+$; n = 16 per group), (**u**) M1-like macrophages (CD206$^-$ within F4/80$^+$CD11b$^+$; n = 14 per group) and (**v**) M2-like macrophages (CD206$^+$ within F4/80$^+$CD11b$^+$; n = 14 per group) in the indicated mouse cSCCs. All data are represented as the mean ± SD, and *n* values indicate independent tumors (**b-v**). **w-y**, mRNA expression levels (mean ± SD) of the indicated genes in (**w**) M-MDSCs, (**x**) M1-like macrophages and (**y**) M2-like macrophages isolated by FACS from mesenchymal mouse cSCCs relative to those from epithelial mouse cSCCs (n = 3 biologically independent samples per group). *P* values determined by one-way ANOVA with Tukey's (**b-v**) multiple comparison test, unpaired two-sided Student's *t*-test (**w-y**). See Supplementary Fig. 2 for gating strategy (**b-v**). Source data are provided as a Source Data file.

Supplementary Fig. 2

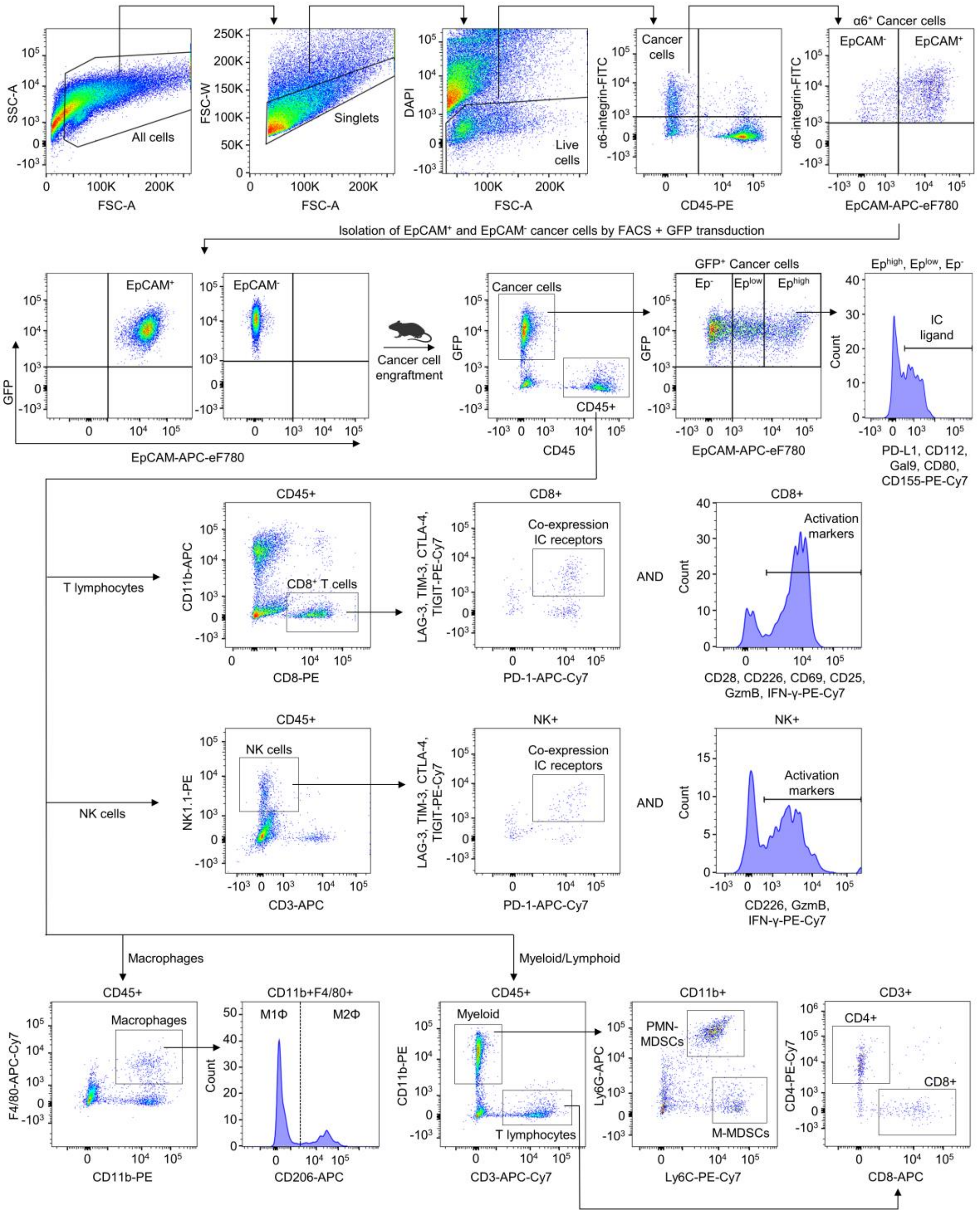

Supplementary Figure 2. Flow cytometry gating strategies for cancer and immune cell populations. Cells were selected and gated to exclude doublets and dead cells. To characterize the changes of cancer cell features during mouse cSCC progression, epithelial EpCAM$^+$ and mesenchymal EpCAM$^-$ cancer cells were isolated by FACS from the α6-integrin$^+$CD45$^-$ cancer cell compartment at different cSCC stages, which had previously been generated by orthotopic serial engraftments[8]. After isolation, α6-integrin$^+$ cancer cells were stably transduced with GFP. To generate cSCCs, epithelial GFP$^+$EpCAM$^+$ and mesenchymal GFP$^+$EpCAM$^-$ cancer cells were

sorted and engrafted into immunocompetent syngeneic mice. Gating strategy highlighting the identification of epithelial GFP^+EpCAM^+ (including GFP^+EpCAM^{high} and GFP^+EpCAM^{low} cells) and mesenchymal GFP^+EpCAM^- cancer cells within the GFP^+CD45^- cancer cell compartment. PD-L1, CD112, Gal9, CD80 and CD155 are gated within $EpCAM^{high}$, $EpCAM^{low}$ and $EpCAM^-$ cancer cells. Gating strategy used to define T lymphocytes ($CD45^+CD3^+CD11b^-$), $CD8^+$ T cells ($CD45^+CD3^+CD8^+CD4^-$) and NK cells ($CD45^+CD3^-NK1.1^+$). PD-1, LAG-3, TIM-3, CTLA-4, TIGIT, CD28, CD226, CD69, CD25, GzmB and IFN-γ are gated within $CD8^+$ T cells and NK cells. Myeloid cells are identified by gating the $CD45^+CD11b^+CD3^-$ population. Within the myeloid compartment, gating strategy used to identify macrophages ($CD45^+CD11b^+F4/80^+$), M1-like macrophages ($CD11b^+F4/80^+CD206^-$), M2-like macrophages ($CD11b^+F4/80^+CD206^+$), PMN-MDSCs ($CD11b^+Ly6C^{lo}Ly6G^+$) and M-MDSCs ($CD11b^+Ly6C^{hi}Ly6G^-$).

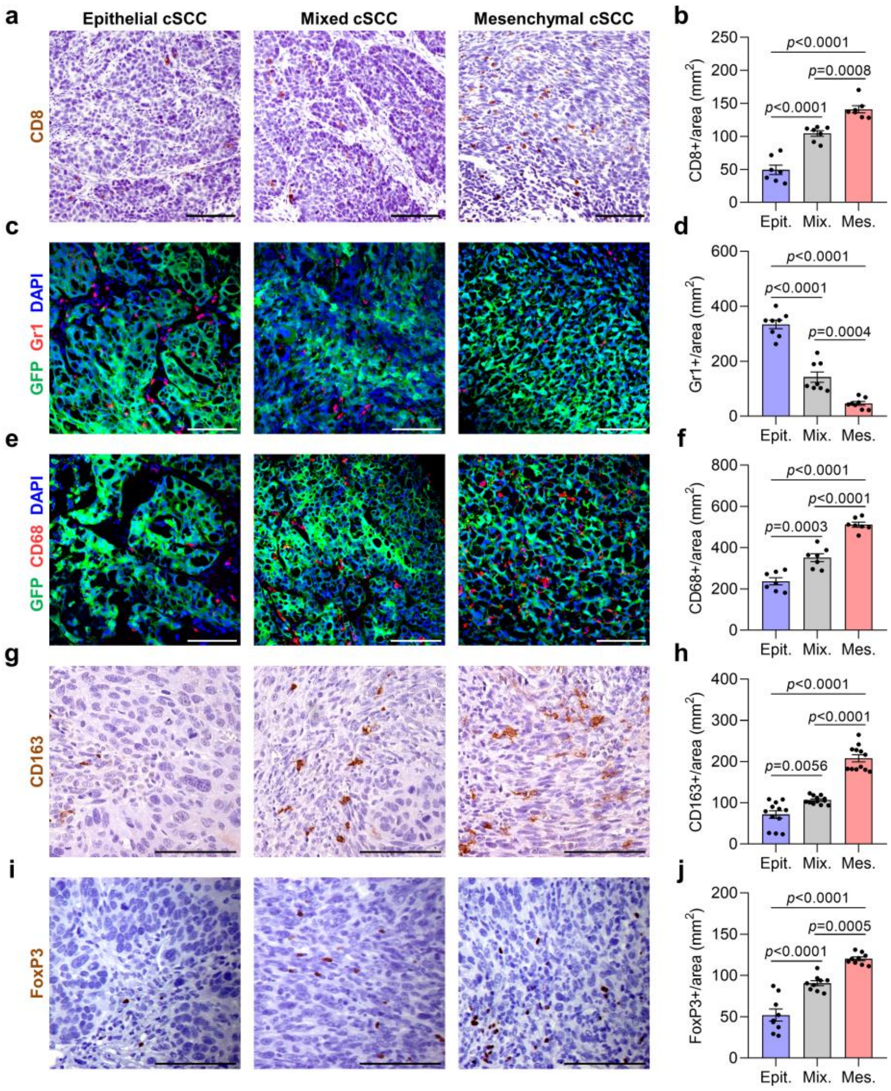

Supplementary Figure 3. Cancer cell features affect TME composition in mouse cSCCs. **a**,**g**,**i**, Representative immunohistochemistry images and quantification of (**b**) CD8$^+$ (n = 7 per group), (**h**) CD163$^+$ (n = 12 per group) and (**j**) FoxP3$^+$ (n = 9 per group) cells per tumor area (mm^2) in the indicated mouse cSCCs. **c**,**e**, Representative immunofluorescence images of GFP$^+$ cancer cells (green), (**c**) Gr1$^+$ or (**e**) CD68$^+$ (red), and DAPI nuclear (blue) staining, and quantification of (**d**) Gr1$^+$ (n = 8 per group) and (**f**) CD68$^+$ (n = 7 per group) cells per tumor area (mm^2) in epithelial, mixed and mesenchymal mouse cSCCs. Scale bar, 100 µm. Each dot indicates the average of at least 5 fields from different tumor regions. All data are represented as the mean ± SEM, and *n* values indicate independent tumors. *P* values determined by one-way ANOVA with Tukey's multiple comparison test. Source data are provided as a Source Data file.

Supplementary Fig. 4

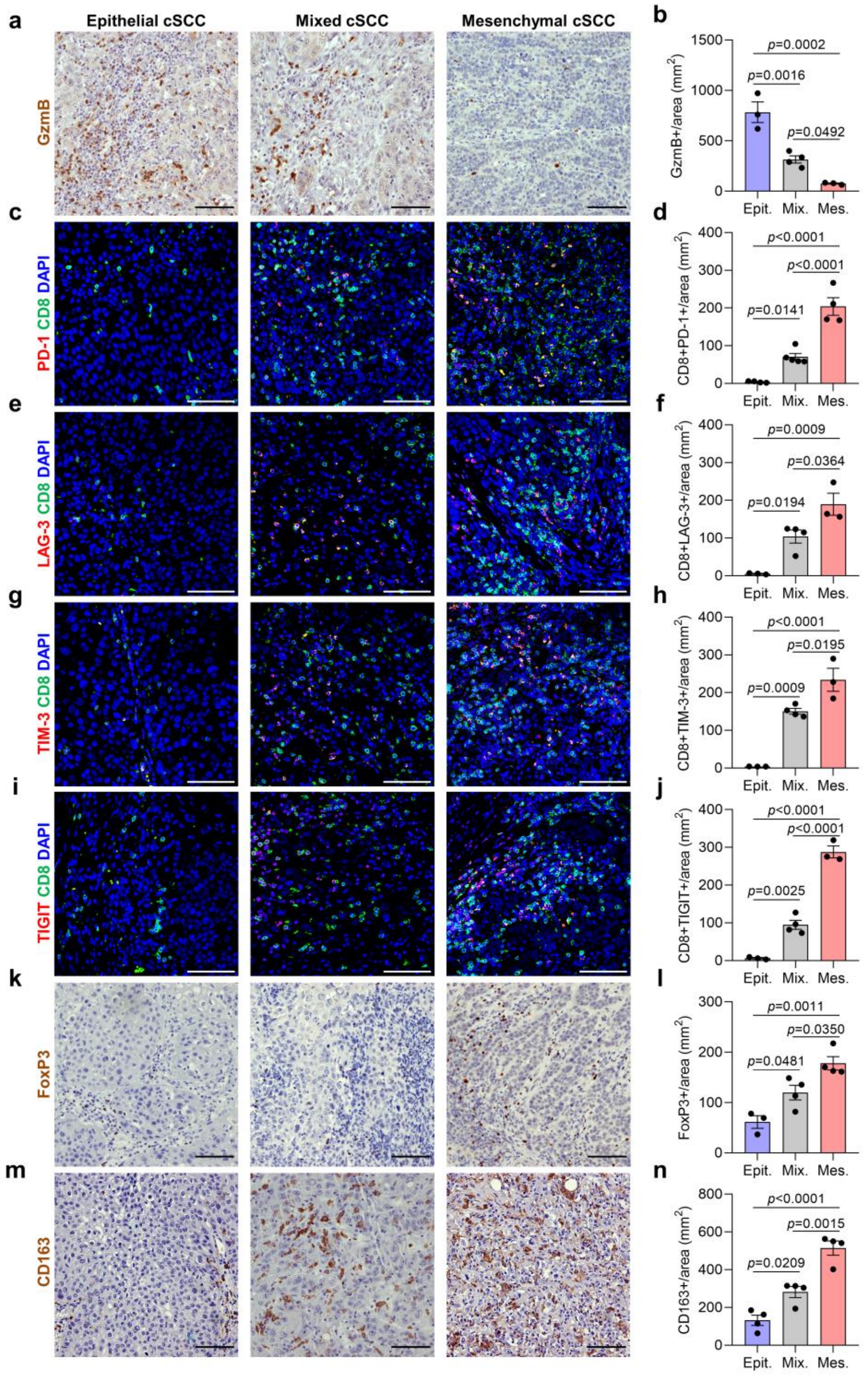

Supplementary Figure 4. Patient cSCCs enriched in hybrid E/M and mesenchymal cancer cells show elevated recruitment of exhausted and immunosuppressive immune cells. a,**k**,**m**, Representative immunohistochemistry images of (**a**) $GzmB^+$, (**k**) $FoxP3^+$ and (**m**) $CD163^+$ cells in the indicated patient cSCCs. Scale bar, 100 µm. **b**, Quantification of $GzmB^+$ cells per tumor area (mm^2) in epithelial (n = 3), mixed (n = 4) and mesenchymal (n = 3) patient cSCCs. **l**, Quantification of $FoxP3^+$ cells per tumor area (mm^2) in epithelial (n = 3), mixed (n = 4) and mesenchymal (n = 4) patient cSCCs. **n**, Quantification of $CD163^+$ cells per tumor area (mm^2) in

epithelial, mixed and mesenchymal patient cSCCs (n = 4 per group). **c**,**e**,**g**,**i**, Representative immunofluorescence images of $CD8^+$ (green), (**c**) $PD\text{-}1^+$, (**e**) $LAG\text{-}3^+$, (**g**) $TIM\text{-}3^+$ or (**i**) $TIGIT^+$ (red), and DAPI nuclear (blue) staining in the indicated patient cSCCs. Scale bar, 100 µm. **d**, Quantification of the frequency of $CD8^+PD\text{-}1^+$ cells per tumor area (mm^2) in epithelial (n = 4), mixed (n = 5) and mesenchymal (n = 4) patient cSCCs. **f**,**h**,**j**, Quantification of the frequency of (**f**) $CD8^+LAG\text{-}3^+$, (**h**) $CD8^+TIM\text{-}3^+$ and (**j**) $CD8^+TIGIT^+$ cells per tumor area (mm^2) in epithelial (n = 3), mixed (n = 4) and mesenchymal (n = 3) patient cSCCs. Each dot indicates the average of at least 5 fields from different tumor regions. All data are represented as the mean ± SEM, and *n* values indicate independent tumors. *P* values determined by one-way ANOVA with Tukey's multiple comparison test. Source data are provided as a Source Data file.

Supplementary Fig. 5

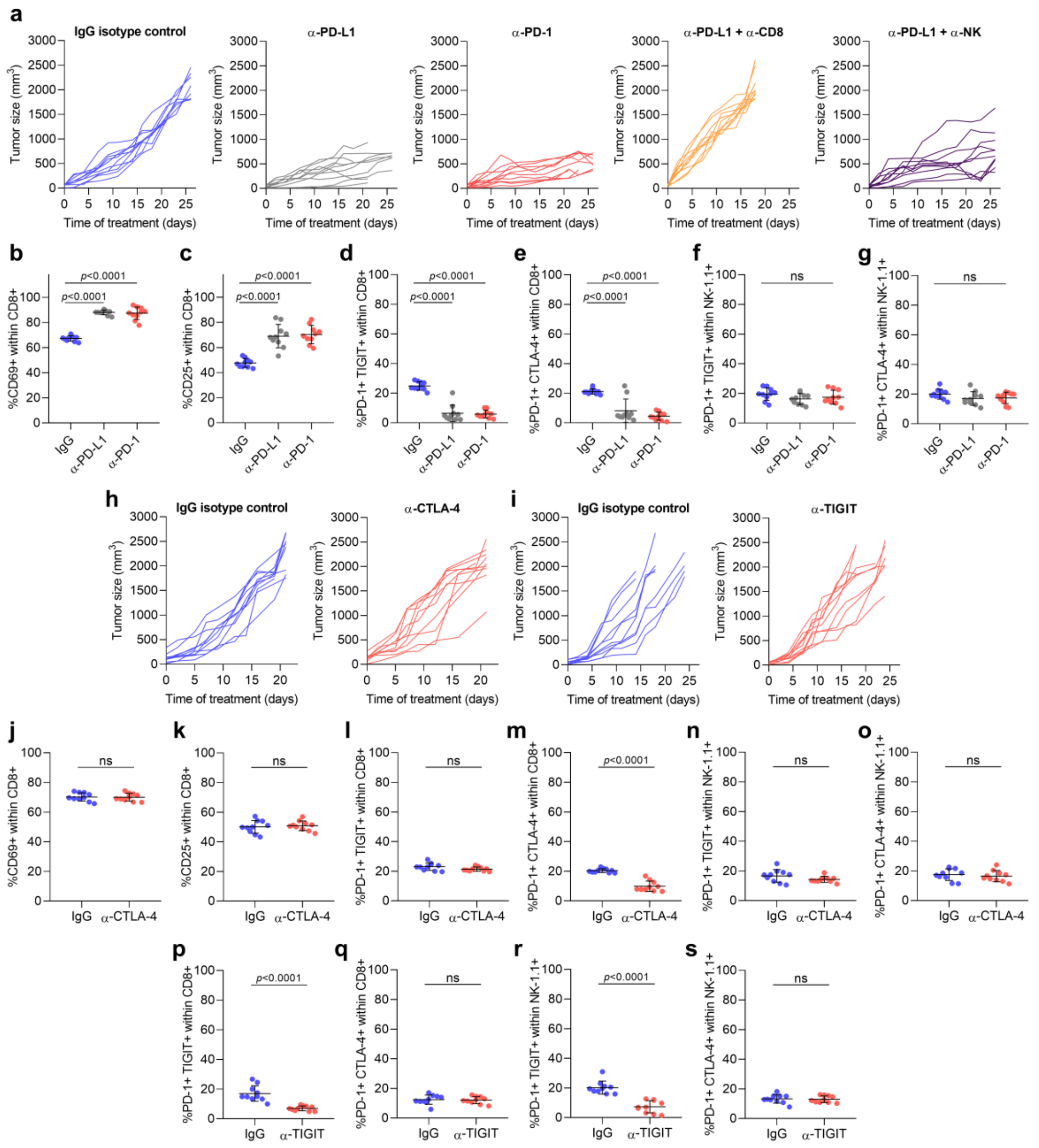

Supplementary Figure 5. Anti-PD-L1/PD-1 response is mediated by CD8$^+$ T cells in mouse epithelial cSCCs. a, Growth kinetics of IgG control, anti-PD-L1, anti-PD-1, anti-PD-L1 + anti-CD8 and anti-PD-L1 + anti-NK-treated epithelial cSCCs (n = 10 per group). Each line represents one single tumor. **b-g**, Percentage of (**b**) CD69$^+$, (**c**) CD25$^+$, (**d**) PD-1$^+$TIGIT$^+$ and (**e**) PD-1$^+$CTLA-4$^+$ cells within CD8$^+$ T cell population, and (**f**) PD-1$^+$TIGIT$^+$ and (**g**) PD-1$^+$CTLA-4$^+$ cells within NK cell population in the indicated epithelial cSCCs (n = 10 per group). **h**,**i**, Growth kinetics of IgG control, (**h**) anti-CTLA-4 and (**i**) anti-TIGIT-treated epithelial cSCCs (n = 10 per group). Each line represents one single tumor. **j-o**, Percentage of (**j**) CD69$^+$, (**k**) CD25$^+$, (**l**) PD-1$^+$TIGIT$^+$ and (**m**) PD-1$^+$CTLA-4$^+$ cells within CD8$^+$ T cell population, and (**n**) PD-1$^+$TIGIT$^+$ and (**o**) PD-1$^+$CTLA-4$^+$ cells within NK cell population in IgG control and anti-CTLA-4-treated epithelial cSCCs (n = 10 per group). **p-s**, Percentage of (**p**) PD-1$^+$TIGIT$^+$ and (**q**) PD-1$^+$CTLA-4$^+$

cells within $CD8^+$ T cell population, and (**r**) PD-1^+TIGIT$^+$ and (**s**) PD-1^+CTLA-4^+ cells within NK cell population in IgG control and anti-TIGIT-treated epithelial cSCCs (n = 10 per group). All data are represented as the mean ± SD, and *n* values indicate independent tumors. *P* values determined by one-way ANOVA with Dunnett's multiple comparison test (**b-g**), unpaired two-sided Student's *t*-test (**j-s**). ns > 0.05: not significant. See Supplementary Fig. 2 for gating strategy (**b-g**,**j-s**). Source data are provided as a Source Data file.

Supplementary Fig. 6

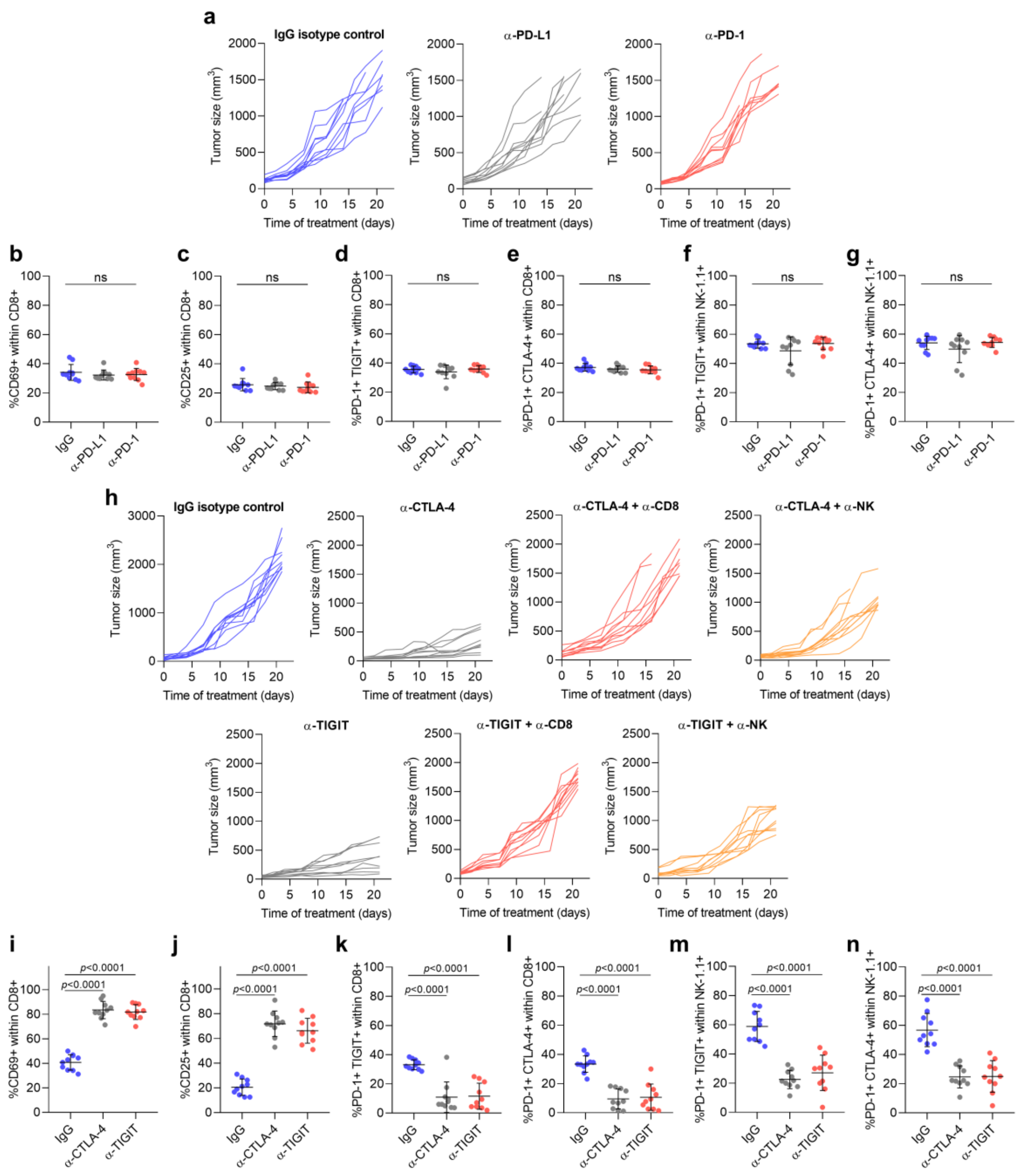

Supplementary Figure 6. Anti-CTLA-4 and anti-TIGIT responses are mediated by CD8$^+$ and NK cells in mouse mesenchymal cSCCs. a, Growth kinetics of IgG control, anti-PD-L1 and anti-PD-1-treated mesenchymal cSCCs (n = 10 per group). Each line represents one single tumor. **b-g**, Percentage of (**b**) CD69$^+$, (**c**) CD25$^+$, (**d**) PD-1$^+$TIGIT$^+$ and (**e**) PD-1$^+$CTLA-4$^+$ cells within CD8$^+$ T cell population, and (**f**) PD-1$^+$TIGIT$^+$ and (**g**) PD-1$^+$CTLA-4$^+$ cells within NK cell population in the indicated mesenchymal cSCCs (n = 10 per group). **h**, Growth kinetics of IgG control, anti-CTLA-4, anti-CTLA-4 + anti-CD8, anti-CTLA-4 + anti-NK, anti-TIGIT, anti-TIGIT + anti-CD8 and anti-TIGIT + anti-NK-treated mesenchymal cSCCs (n = 10 per group). Each line represents one single tumor. **i-n**, Percentage of (**i**) CD69$^+$, (**j**) CD25$^+$, (**k**) PD-1$^+$TIGIT$^+$ and (**l**) PD-1$^+$CTLA-4$^+$ cells within CD8$^+$ T cell population, and (**m**) PD-1$^+$TIGIT$^+$ and (**n**) PD-1$^+$CTLA-

4$^+$ cells within NK cell population in the indicated mesenchymal cSCCs (n = 10 per group). All data are represented as the mean ± SD, and *n* values indicate independent tumors. *P* values determined by one-way ANOVA with Dunnett's multiple comparison test. ns > 0.05: not significant. See Supplementary Fig. 2 for gating strategy (**b-g**,**i-n**). Source data are provided as a Source Data file.

Supplementary Fig. 7

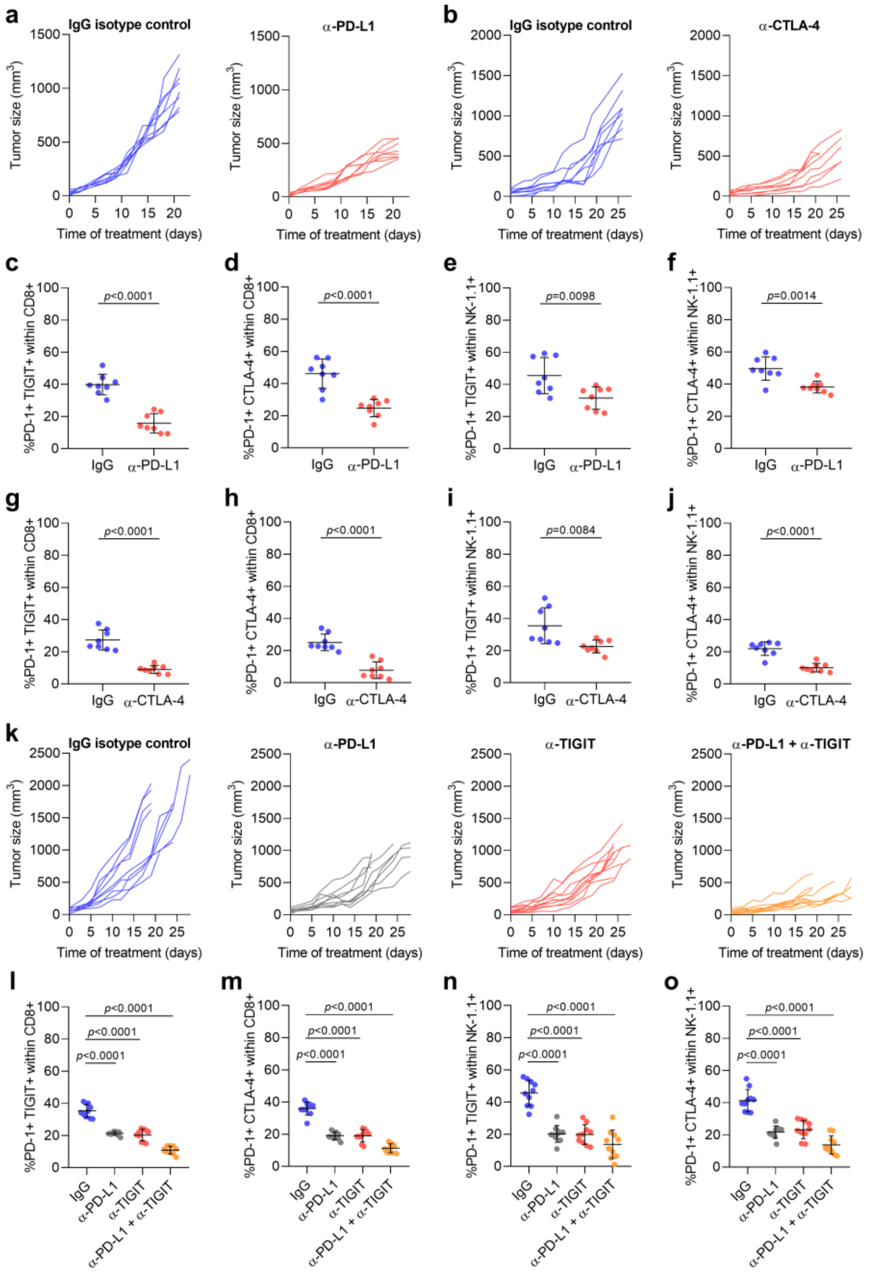

Supplementary Figure 7. Combined anti-PD-L1 and anti-TIGIT therapies suppress mixed mouse cSCC growth by targeting epithelial and mesenchymal cancer cells. a,**b**, Growth kinetics of IgG control, (**a**) anti-PD-L1 and (**b**) anti-CTLA-4-treated mixed cSCCs (n = 8 per group). Each line represents one single tumor. **c**-**j**, Percentage of (**c**,**g**) PD-1^{+}TIGIT^{+} and (**d**,**h**) PD-1^{+}CTLA-4^{+} cells within CD8^{+} T cell population, and (**e**,**i**) PD-1^{+}TIGIT^{+} and (**f**,**j**) PD-1^{+}CTLA-4^{+} cells within NK cell population in IgG control, anti-PD-L1 and anti-CTLA-4-treated mixed cSCCs (n = 8 per group). **k**, Growth kinetics of IgG control, anti-PD-L1, anti-TIGIT and anti-PD-L1 + anti-TIGIT-treated mixed cSCCs (n = 10 per group). Each line represents one single tumor. **l**-**o**, Percentage of (**l**) PD-1^{+}TIGIT^{+} and (**m**) PD-1^{+}CTLA-4^{+} cells within CD8^{+} T cell population, and (**n**) PD-1^{+}TIGIT^{+} and (**o**) PD-1^{+}CTLA-4^{+} cells within NK cell population in IgG

control, anti-PD-L1, anti-TIGIT and anti-PD-L1 + anti-TIGIT-treated mixed cSCCs (n = 10 per group). All data are represented as the mean ± SD, and *n* values indicate independent tumors. *P* values determined by unpaired two-sided Student's *t*-test (**c-j**), one-way ANOVA with Dunnett's multiple comparison test (**l-o**). See Supplementary Fig. 2 for gating strategy (**c-j**,**l-o**). Source data are provided as a Source Data file.

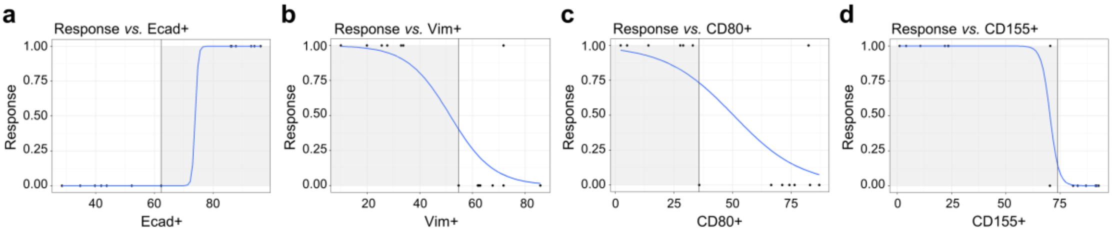

e

cSCC	E-cadherin Estim	E-cadherin Low.lim (95%)	E-cadherin Up.lim (95%)	Vimentin Estim	Vimentin Low.lim (95%)	Vimentin Up.lim (95%)	CD80 Estim	CD80 Low.lim (95%)	CD80 Up.lim (95%)	CD155 Estim	CD155 Low.lim (95%)	CD155 Up.lim (95%)
Sensitivity	0.857	0.421	0.996	1	0.59	1	1	0.59	1	0.857	0.421	0.996
Specificity	1	0.59	1	0.857	0.421	0.996	0.857	0.421	0.996	1	0.59	1

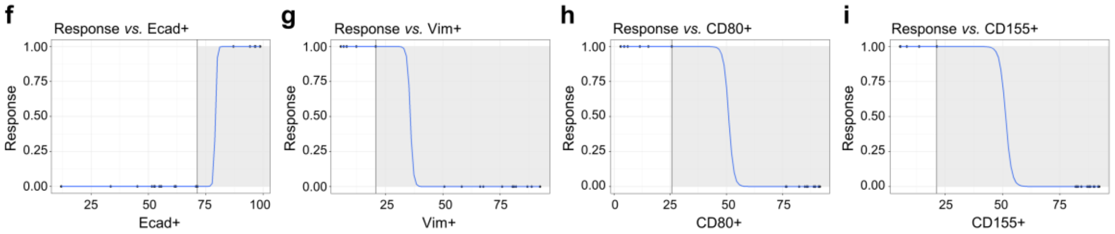

j

HNSCC	E-cadherin Estim	E-cadherin Low.lim (95%)	E-cadherin Up.lim (95%)	Vimentin Estim	Vimentin Low.lim (95%)	Vimentin Up.lim (95%)	CD80 Estim	CD80 Low.lim (95%)	CD80 Up.lim (95%)	CD155 Estim	CD155 Low.lim (95%)	CD155 Up.lim (95%)
Sensitivity	0.923	0.64	0.998	1	0.753	1	1	0.753	1	1	0.753	1
Specificity	1	0.541	1	0.833	0.359	0.996	0.833	0.359	0.996	0.833	0.359	0.996

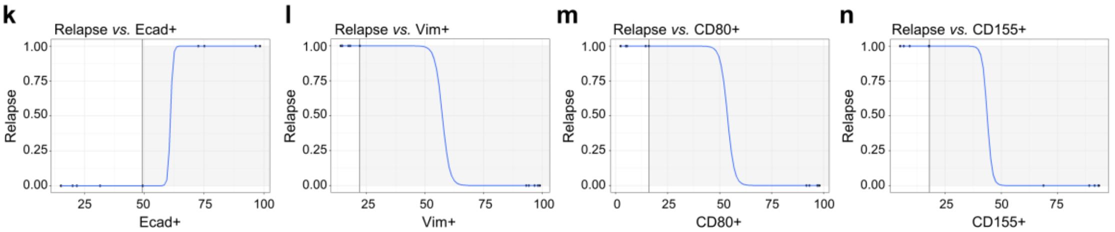

o

Melanoma	E-cadherin Estim	E-cadherin Low.lim (95%)	E-cadherin Up.lim (95%)	Vimentin Estim	Vimentin Low.lim (95%)	Vimentin Up.lim (95%)	CD80 Estim	CD80 Low.lim (95%)	CD80 Up.lim (95%)	CD155 Estim	CD155 Low.lim (95%)	CD155 Up.lim (95%)
Sensitivity	0.8	0.284	0.995	1	0.478	1	1	0.478	1	1	0.478	1
Specificity	1	0.478	1	0.8	0.284	0.995	0.8	0.284	0.995	0.8	0.284	0.995

Supplementary Figure 8. Anti-PD-1/PD-L1 resistance in cSCC, HNSCC and melanoma patient samples is associated with a higher frequency of hybrid E/M and mesenchymal cancer cells. a-d, Smoothed logistic regression curves with 95% confidence interval (CI) relating the percentage of (**a**) Ecad$^+$, (**b**) Vim$^+$, (**c**) CD80$^+$ and (**d**) CD155$^+$ cancer cells to the response to anti-PD-1/PD-L1 therapy in cSCC patient samples (0 = non-response; 1 = response). **f-i**, Smoothed logistic regression curves with 95% CI relating the percentage of (**f**) Ecad$^+$, (**g**) Vim$^+$, (**h**) CD80$^+$ and (**i**) CD155$^+$ cancer cells to the response to anti-PD-1/PD-L1 therapy in HNSCC patient samples (0 = non-response; 1 = response). **k-n**, Smoothed logistic regression curves with

95% CI relating the percentage of (**k**) Ecad$^+$, (**l**) Vim$^+$, (**m**) CD80$^+$ and (**n**) CD155$^+$ cancer cells to the probability of relapse during adjuvant anti-PD-1 therapy in melanoma patient samples (0 = relapse; 1 = non-relapse). The vertical lines represent the best-performing cut-off point for each variable. **a**-**d**, Anti-PD-1/PD-L1 responder and non-responder cSCCs (n = 7 per group); **f**-**i**, anti-PD-1/PD-L1 responder and non-responder HNSCCs (n = 6 responders, n = 13 non-responders); **k**-**n**, anti-PD-1 non-relapsed and relapsed melanomas (n = 5 per group). **e**,**j**,**o**, Diagnostic accuracy measures for the cut-off point obtained for each variable below (Ecad) or above (Vim, CD80 and CD155) which it could be considered a risk factor for showing anti-PD-1/PD-L1 resistance.

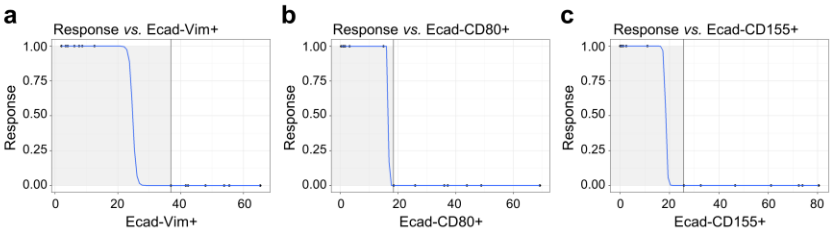

d

	Ecad-Vim+			Ecad-CD80+			Ecad-CD155+		
**cSCC**	Estim	Low.lim (95%)	Up.lim (95%)	Estim	Low.lim (95%)	Up.lim (95%)	Estim	Low.lim (95%)	Up.lim (95%)
Sensitivity	1	0.59	1	1	0.59	1	1	0.59	1
Specificity	1	0.59	1	1	0.59	1	1	0.59	1

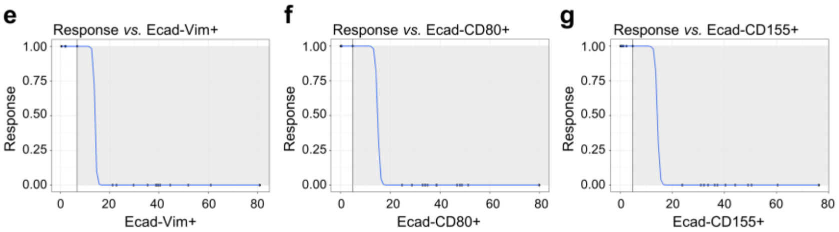

h

	Ecad-Vim+			Ecad-CD80+			Ecad-CD155+		
**HNSCC**	Estim	Low.lim (95%)	Up.lim (95%)	Estim	Low.lim (95%)	Up.lim (95%)	Estim	Low.lim (95%)	Up.lim (95%)
Sensitivity	1	0.753	1	1	0.753	1	1	0.753	1
Specificity	0.833	0.359	0.996	0.833	0.359	0.996	0.833	0.359	0.996

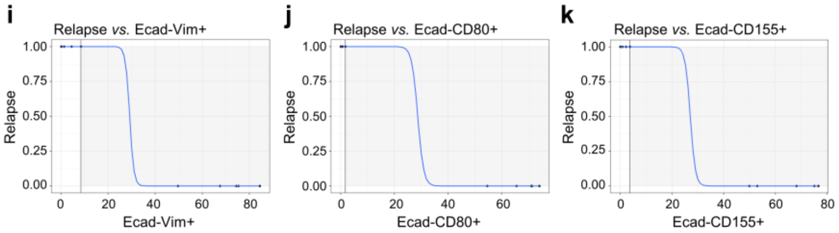

l

	Ecad-Vim+			Ecad-CD80+			Ecad-CD155+		
**Melanoma**	Estim	Low.lim (95%)	Up.lim (95%)	Estim	Low.lim (95%)	Up.lim (95%)	Estim	Low.lim (95%)	Up.lim (95%)
Sensitivity	1	0.478	1	1	0.478	1	1	0.478	1
Specificity	0.8	0.284	0.995	0.8	0.284	0.995	0.8	0.284	0.995

Supplementary Figure 9. Anti-PD-1/PD-L1 non-responder and relapsed samples have a higher frequency of mesenchymal $Ecad^-Vim^+$, $Ecad^-CD80^+$ and $Ecad^-CD155^+$ cancer cells than responder and non-relapsed samples. a-c, Smoothed logistic regression curves with 95% confidence interval (CI) relating the percentage of (**a**) $Ecad^-Vim^+$, (**b**) $Ecad^-CD80^+$ and (**c**) $Ecad^-CD155^+$ cancer cells to the response to anti-PD-1/PD-L1 therapy in cSCC patient samples (0 = non-response; 1 = response). **e-g**, Smoothed logistic regression curves with 95% CI relating the percentage of (**e**) $Ecad^-Vim^+$, (**f**) $Ecad^-CD80^+$ and (**g**) $Ecad^-CD155^+$ cancer cells to the response to anti-PD-1/PD-L1 therapy in HNSCC patient samples (0 = non-response; 1 = response). **i-k**,

Smoothed logistic regression curves with 95% CI relating the percentage of (**i**) $Ecad^{-}Vim^{+}$, (**j**) $Ecad^{-}CD80^{+}$ and (**k**) $Ecad^{-}CD155^{+}$ cancer cells to the probability of relapse during adjuvant anti-PD-1 therapy in melanoma patient samples (0 = relapse; 1 = non-relapse). The vertical lines represent the best-performing cut-off point for each variable. **a-c**, Anti-PD-1/PD-L1 responder and non-responder cSCCs (n = 7 per group); **e-g**, anti-PD-1/PD-L1 responder and non-responder HNSCCs (n = 6 responders, n = 13 non-responders); **i-k**, anti-PD-1 non-relapsed and relapsed melanomas (n = 5 per group). **d**,**h**,**l**, Diagnostic accuracy measures for the cut-off point obtained for each variable above which it could be considered a risk factor for showing anti-PD-1/PD-L1 resistance.

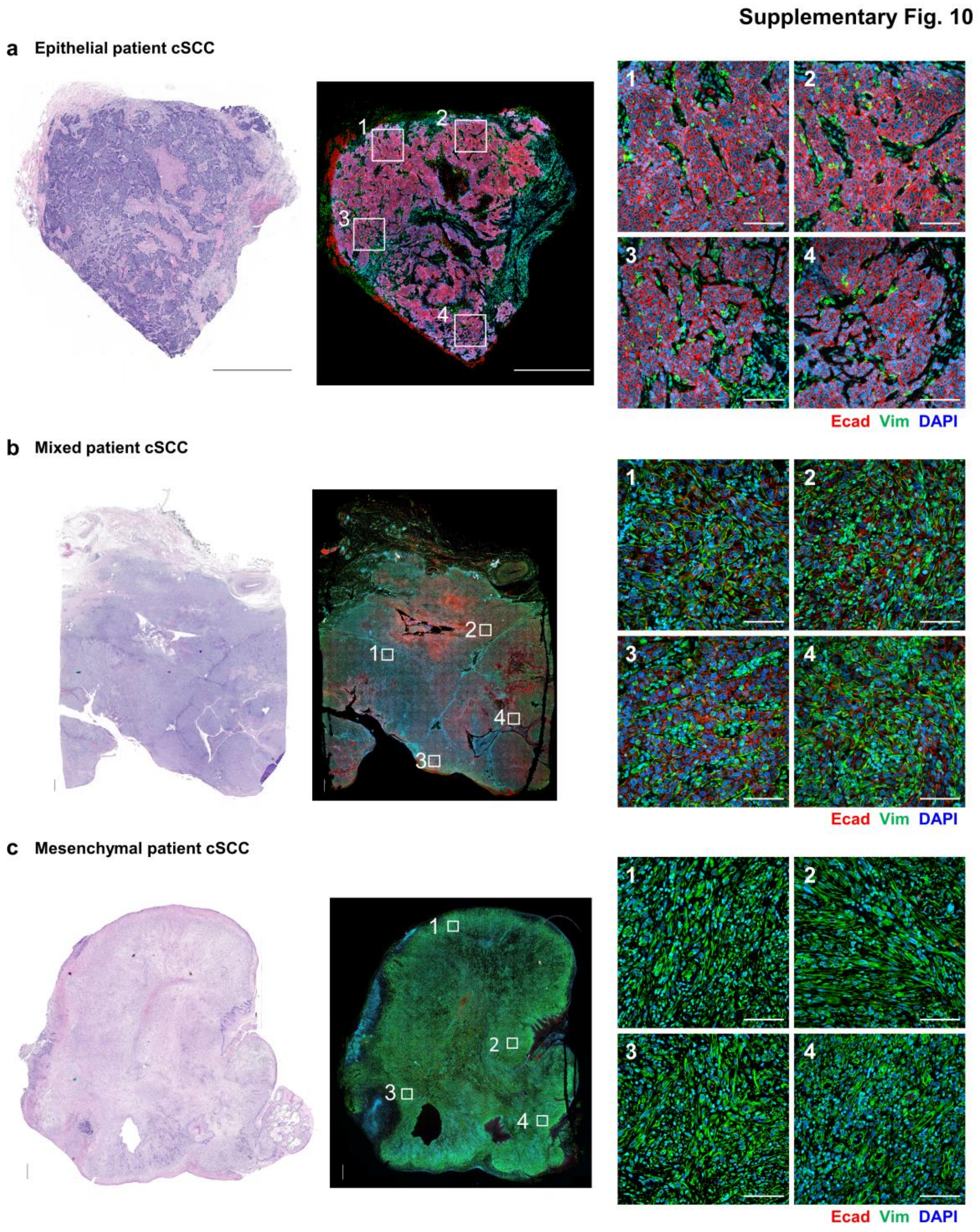

Supplementary Figure 10. Epithelial, hybrid E/M and mesenchymal cancer cells are detected in cSCC patient samples. a-c, Representative hematoxylin/eosin (H/E) and immunofluorescence images of $Ecad^+$ (red), Vim^+ (green) and DAPI nuclear (blue) staining in an (**a**) epithelial (H49, representative of n = 4 tumors), (**b**) mixed (H48, representative of n = 6 tumors) and (**c**) mesenchymal (H15, representative of n = 4 tumors) patient cSCC. Boxed images enlarged to the right of each cSCC. Scale bar, 1000 µm (main images), 100 µm (magnified images).

	Patient	Gender	Primary tumor site	Disease extent	Previous systemic therapy	Best overall response[a]	Progressive disease (PD)	Exitus	Cause of exitus	DoR (months)	Time to PD (months)	OS (months)
Responders	P1	Male	Scalp	Metastatic	None	CR	No	No	-	36.8	-	44.1
	P2	Female	Neck	Metastatic	None	PR	No	No	-	36.0	-	41.6
	P3	Male	Scalp	Unresectable locally adv.	None	PR	Yes	No	-	18.3	20.2	25.7
	P4	Male	Head	Metastatic	CBDCA + 5-FU	PR	No	Yes	Pneumonia	22.4	Not reached	24.2
	P5	Female	Leg	Metastatic	None	PR	Yes	No	-	21.5	22.9	27.5
	P6	Male	Leg	Metastatic	None	PR	No	Yes	Clinical impairment	6.7	Not reached	7.9
	P7	Male	Head	Metastatic	None	PR	No	No	-	16.4	-	20.9
									Median	21.5	21.5	25.7
Non-responders	P8	Female	Head	Unresectable locally adv.	None	SD	No	No	-	-	-	9.5
	P9	Male	Head	Unresectable locally adv.	Cetuximab	SD	Yes	Yes	Sepsis	-	8.9	22.7
	P10	Female	Head	Metastatic	None	PD	Yes	Yes	PD	-	1.1	1.9
	P11	Male	Neck	Metastatic	Cisplatin	PR	Yes	Yes	PD	2.6	4.4	22.2
	P12	Male	Leg	Metastatic	Cisplatin	PD	Yes	Yes	PD	-	1.6	1.6
	P13	Male	Head	Metastatic	None	PD	Yes	No	-	-	2.5	24.3
	P14	Female	Head	Metastatic	None	PD	Yes	Yes	PD	-	1.5	1.5
									Median	-	2.1	9.5

Supplementary Table 1. Clinical features of anti-PD-1/PD-L1 responder and non-responder cSCC patients. [a]Tumor response assessed using RECIST v1.1. We considered patients as responders if they achieved complete or partial response as best response for at least 3 months, while non-responder patients are those who had stable or progressive disease as their best response. Patient P11 is classified as a non-responder because he showed a PR for less than 3 months. The best overall response is the best response recorded from the start of treatment until the end of treatment. Duration of response (DoR) is defined as the time between the date of first response assessment showing CR or PR and the date of progressive disease or exitus; time to progression is defined as the time between the start of treatment and the date of progressive disease; overall survival (OS) is defined as the time between the start of treatment and the date of exitus from any cause. “Not reached” is considered when a patient dies without having exhibited progressive disease. All patients are immunocompetent. CR: complete response; PR: partial response; SD: stable disease; PD: progressive disease; CBDCA: carboplatin; 5-FU: 5-fluorouracil.

	Patient	Gender	Primary tumor site	Cancer staging	PD-L1 CPS	Best overall response[a]	Progressive disease (PD)	Exitus	Cause of exitus	DoR (months)	Time to PD (months)	OS (months)
Responders	P1	Male	Hypopharynx	IVC	20	PR	No	No	-	11.7	-	16.2
	P2	Male	Larynx	IVB	40	PR	Yes	Yes	PD	12.0	19.6	20.5
	P3	Male	Larynx	IVA	<50	CR	Yes	No	-	35.7	46.5	54.0
	P4	Male	Larynx	III	0	PR	No	Yes	Hemoptysis	22.5	Not reached	28.6
	P5	Male	Larynx	IVC	0	PR	Yes	Yes	PD	19.5	21.2	27.8
	P6	Male	Larynx	III	90	PR	Yes	No	-	6.9	10.8	12.4
									Median	15.7	20.4	24.2
Non-responders	P7	Female	Oral cavity	IVA	25	PD	Yes	No	-	-	2.1	19.6
	P8	Female	Oral cavity	IVA	35	PD	Yes	Yes	PD	-	3.4	3.9
	P9	Male	Oral cavity	IVB	95	PD	Yes	Yes	PD	-	0.7	0.8
	P10	Female	Sinus	IVA	0	PD	Yes	Yes	PD	-	1.3	6.3
	P11	Male	Oral cavity	IVB	0	PD	Yes	Yes	PD	-	2.4	4.0
	P12	Male	Oral cavity	IVA	<50	PD	Yes	No	-	-	2.2	66.7
	P13	Male	Hypopharynx	IVA	2	PD	Yes	Yes	PD	-	0.9	1.5
	P14	Male	Larynx	IVA	0	PD	Yes	Yes	PD	-	2.0	4.0
	P15	Male	Oropharynx	IVC	0	PD	Yes	Yes	PD	-	1.0	3.6
	P16	Male	Larynx	IVA	0	PD	Yes	Yes	PD	-	1.4	2.0
	P17	Male	Oral cavity	IVA	0	PD	Yes	Yes	PD	-	2.2	5.1
	P18	Male	Larynx	III	0	PD	Yes	Yes	PD	-	1.4	7.7
	P19	Male	Larynx	III	0	PD	Yes	Yes	PD	-	1.1	7.2
									Median	-	1.4	4.0

Supplementary Table 2. Clinical features of anti-PD-1/PD-L1 responder and non-responder HNSCC patients. [a]Tumor response assessed using RECIST v1.1. We considered patients as responders if they achieved complete or partial response as best response for at least 3 months, while non-responder patients are those who had progressive disease as their best response. The best overall response is the best response recorded from the start of treatment until the end of treatment. Duration of response (DoR) is defined as the time between the date of first response assessment showing CR or PR and the date of progressive disease or exitus; time to progression is defined as the time between the start of treatment and the date of progressive disease; overall survival (OS) is defined as the time between the start of treatment and the date of exitus from any cause. “Not reached” is considered when a patient dies without having exhibited progressive disease. CR: complete response; PR: partial response; PD: progressive disease; CPS: combined positive score.

Group	Patient	Gender	Primary tumor site	Cancer staging	Relapse	Exitus	Cause of exitus	Time to relapse (months)	OS (months)
Non-relapsed	P1	Male	Skin	IIIC	Yes	Yes	PD	33.8	48.9
	P2	Male	Skin	IIIC	No	No	-	-	28.2
	P3	Male	Skin	IIIC	No	No	-	-	26.6
	P4	Male	Skin	IIIC	No	No	-	-	37.5
	P5	Male	Skin	IIIC	No	No	-	-	27.4
							Median	33.8	28.2
Relapsed	P6	Male	Skin	IIIC	Yes	Yes	PD	6.8	18.2
	P7	Male	Skin	IIIC	Yes	No	-	9.4	57.8
	P8	Male	Skin	IIIC	Yes	Yes	PD	6.3	16.7
	P9	Female	Skin	IIIC	Yes	Yes	PD	7.8	26.0
	P10	Female	Skin	IIIC	Yes	Yes	PD	10.1	36.2
							Median	7.8	26.0

Supplementary Table 3. Clinical features of anti-PD-1 non-relapsed and relapsed melanoma patients. We considered patients as non-relapsed if they did not relapse within 18 months of starting adjuvant anti-PD-1 treatment, while relapsed patients were those who relapsed within that period. Time to relapse is defined as the time between the start of adjuvant anti-PD-1 treatment and the date of relapse; overall survival (OS) is defined as the time between the start of adjuvant anti-PD-1 treatment and the date of exitus from any cause. PD: progressive disease.

Antibody	Clone	Commercial reference	Dilution
Anti-mouse/human CD11b-APC	M1/70	Biolegend, Cat: 101211	1:250 (FC)
Anti-mouse/human CD11b-PE	M1/70	BD Bioscience, Cat: 557397	1:250 (FC)
Anti-mouse/human CD11b PE/Cy7	M1/70	Biolegend, Cat: 101215	1:250 (FC)
Anti-mouse CD152 (CTLA-4)-PE/Cy7	UC10-4B9	Biolegend, Cat: 106313	1:250 (FC)
Anti-mouse CD155 (PVR)-PE/Cy7	TX56	Biolegend, Cat: 131511	1:200 (FC)
Anti-mouse CD206 (MMR)-APC	MR6F3	eBioscience, Cat: 17-2061-80	1:200 (FC)
Anti-mouse CD223 (LAG-3)-PE/Cy7	C9B7W	Biolegend, Cat: 125225	1:250 (FC)
Anti-mouse CD226 (DNAM-1)-PE/Cy7	10E5	Biolegend, Cat: 128811	1:250 (FC)
Anti-mouse CD25-PE/Cy7	PC61	Biolegend, Cat: 102015	1:200 (FC)
Anti-mouse CD274 (PD-L1)-PE/Cy7	10F.9G2	Biolegend, Cat: 124313	1:200 (FC)
Anti-mouse CD279 (PD-1)-APC/Cy7	29F.1A12	Biolegend, Cat: 135223	1:250 (FC)
Anti-mouse CD28-PE/Cy7	37.51	Biolegend, Cat: 102125	1:250 (FC)
Anti-mouse CD3ε-APC	145-2C11	Biolegend, Cat: 100311	1:200 (FC)
Anti-mouse CD326 (Ep)-APC-eF780	G8.8	eBioscience, Cat: 47-5791-82	1:400 (FC)
Anti-mouse CD366 (TIM-3)-PE/Cy7	B8.2C12	Biolegend, Cat: 134009	1:250 (FC)
Anti-mouse CD4-PE/Cy7	RM4-5	Biolegend, Cat: 100528	1:200 (FC)
Anti-mouse CD45-PE	30-F11	TONBO, Cat: 50-0451	1:350 (FC)
Anti-mouse/human CD49f-FITC	GoH3	Biolegend, Cat: 313605	1:10 (FC)
Anti-mouse CD69-PE/Cy7	H1.2F3	Biolegend, Cat: 104511	1:200 (FC)
Anti-mouse CD8a-PE	53-6.7	Biolegend, Cat: 100707	1:200 (FC)
Anti-mouse CD80-PE/Cy7	16-10A1	Biolegend, Cat: 104733	1:250 (FC)
Anti-mouse F4/80-APC/Cy7	BM8	Biolegend, Cat: 123118	1:200 (FC)
Anti-mouse Galectin-9-PE/Cy7	08A2	Biolegend, Cat: 137913	1:250 (FC)
Anti-mouse Gr-1-PE/Cy7	RB6-8C5	Biolegend, Cat: 108415	1:250 (FC)
Anti-mouse Granzyme B-PE/Cy7	NGZB	eBioscience, Cat: 25-8898-80	1:200 (FC)
Anti-mouse IFN-γ-PE/Cy7	XMG1.2	Biolegend, Cat: 505825	1:200 (FC)
Anti-mouse Ly-6C-PE/Cy7	HK1.4	Biolegend, Cat: 128017	1:250 (FC)
Anti-mouse Ly-6G-APC	1A8	Biolegend, Cat: 127613	1:250 (FC)
Anti-mouse Nectin-2/CD112-APC	829038	R&D, Cat: FAB3869A	1:200 (FC)
Anti-mouse NK-1.1-PE	PK136	Biolegend, Cat: 108707	1:200 (FC)
Anti-mouse TIGIT (Vstm3)-PE/Cy7	1G9	Biolegend, Cat: 142107	1:250 (FC)
Anti-mouse/human CD163	EPR19518	Abcam, Cat: ab182422	1:50 (IHC)
Anti-mouse CD68	-	Abcam, Cat: ab125212	1:200 (IF)
Anti-mouse CD8α	D4W2Z	Cell Signaling, Cat: 98941	1:50 (IHC)
Anti-mouse FoxP3	D6O8R	Cell Signaling, Cat: 12653	1:50 (IHC)
Anti-mouse Ly-6G/Ly-6C (Gr-1)	RB6-8C5	R&D Systems, Cat: MAB1037	1:200 (IF)
Anti-human CD8α	C8/144B	Abcam, Cat: ab17147	1:50 (IF)
Anti-mouse/human CD80	-	Abcam, Cat: ab254579	1:150 (IF)
Anti-human E-cadherin	36/E-Cadherin	BD Bioscience, Cat: 610182	1:100 (IF)
Anti-human FoxP3	D2W8E	Cell Signaling, Cat: 98377	1:50 (IHC)
Anti-human Granzyme B	-	Abcam, Cat: ab4059	1:100 (IHC)
Anti-human LAG-3	D2G4O	Cell Signaling, Cat: 15372	1:100 (IF)
Anti-human PD-1	EPR4877(2)	Abcam, Cat: ab137132	1:100 (IF)
Anti-human PVR/CD155	D3G7H	Cell Signaling, Cat: 13544	1:100 (IF)

Anti-human TIGIT	E5Y1W	Cell Signaling, Cat: 99567	1:100 (IF)
Anti-human TIM-3	D5D5R	Cell Signaling, Cat: 45208	1:100 (IF)
Anti-human Vimentin	-	Abcam, Cat: ab45939	1:100 (IF)

Supplementary Table 4. Antibodies used for flow cytometry (FC), immunohistochemistry (IHC) and immunofluorescence (IF) assays.

Gene	Forward (5'–3')	Reverse (5'–3')
Arg1	TTTTAGGGTTACGGCCGGTG	CCTCGAGGCTGTCCTTTTGA
Ccl2	GCATCCACGTGTTGGCTCA	CTCCAGCCTACTCATTGGGATCA
Ccl22	ACCTCTGATGCAGGTCCCTA	CTTGCGGCAGGATTTTGAGG
Cdh1	ATCCTCGCCCTGCTGATT	ACCACCGTTCTCCTCCGTA
Cxcl10	ATGACGGGCCAGTGAGAATG	TCGTGGCAATGATCTCAACAC
Cxcl9	GCCATGAAGTCCGCTGTTCT	TAGGGTTCCTCGAACTCCACA
dNp63	GTACCTGGAAAACAATGCCCAG	CGCTATTCTGTGCGTGGTCTG
Epcam	CCGCGGCTCAGAGAGACT	AGGAAGTACACTGGCATTCACC
Fizz1	CCTGCTGGGATGACTGCTAC	CAGTGGTCCAGTCAACGAGT
Gapdh	AGGTCGGTGTGAACGGATTTG	TGTAGACCATGTAGTTGAGGTCA
Gas6	GGAGGCCTGCCAGAAGTATC	TGCTTGTACGAGGCCGTATC
Grhl1	CCTTCACGTGGGACATCAAT	AGCCCTTCACACCCTTCTG
Grhl2	GACAACAAATGCTTCCGACA	GCTGCTCATCTCGGTTTTTG
Il10	GGCGCTGTCATCGATTTCTC	ATGGCCTTGTAGACACCTTGG
Il12b	GCACCAAATTACTCCGGACG	TGGTCCAGTGTGACCTTCTC
Il18	CCTCTTGGCCCAGGAACAAT	ACAGTGAAGTCGGCCAAAGT
Il23a	ACCAGCGGGACATATGAATCT	AGACCTTGGCGGATCCTTTG
Il6	ACCAGAGGAAATTTTCAATAGGC	TGATGCACTTGCAGAAAACA
Krt14	GGCCCAGATCCAGGAGATGAT	CAGGGGCTCTTCCAGCAGTATC
Nos2	TCCTGGACATTACGACCCCT	CTCTGAGGGCTGACACAAGG
Ovol1	CTCCACGTGCAAGAGGAACT	CTCTGGTTCCCGGTAGGG
Ovol2	GCCAGGTCAAAAATCAAGTTTACCA	AGCTCTTGCCACAAAGGTCA
Pdl1	CGCCTGCAGATAGTTCCCAA	AGCCGTGATAGTAAACGCCC
Ptgs2	TGAGTACCGCAAACGCTTCT	CAGCCATTTCCTTCTCTCCTGT
Ppia	GTTCATGCCTTCTTTCACCTTCCC	CAAATGCTGGACCAAACACAAACG
Snai1	CTTGTGTCTGCACGACCTGT	AGTGGGAGCAGGAGAATGG
Tgfb	TGGAGCAACATGTGGAACTC	GTCAGCAGCCGGTTACCA
Tnfa	GCCTCTTCTCATTCCTGCTTG	CTGATGAGAGGGAGGCCATT
Twist	AGCTACGCCTTCTCCGTCT	TCCTTCTCTGGAAACAATGACA
Vegfa	GGCCTCCGAAACCATGAACT	CTGGGACCACTTGGCATGG
Vim	AGAGAGAGGAAGCCGAAAGC	TCCACTTTCCGTTCAAGGTC
Zeb1	GCCAGCAGTCATGATGAAAA	TATCACAATACGGGCAGGTG
Zeb2	TCTTATCAATGAAGCAGCCG	TGCGTCCACTACGTTGTCAT

Supplementary Table 5. Primers used in qRT-PCR assays.
