# Supplementary material for: Cancer cell plasticity defines response to immunotherapy in cutaneous squamous cell carcinoma
Source: Nat Commun. 2024 Jun 24;15:5352. doi: 10.1038/s41467-024-49718-8 (PMC11196727; doi:10.1038/s41467-024-49718-8)
Supplement: Supplementary file 3 — Reporting Summary [file 41467_2024_49718_MOESM3_ESM.pdf]

nature portfolio

Corresponding author(s): Laura Lorenzo-Sanz
Purificación Muñoz

Last updated by author(s): Jun 10, 2024

Reporting Summary

Nature Portfolio wishes to improve the reproducibility of the work that we publish. This form provides structure for consistency and transparency in reporting. For further information on Nature Portfolio policies, see our Editorial Policies and the Editorial Policy Checklist.

Statistics

For all statistical analyses, confirm that the following items are present in the figure legend, table legend, main text, or Methods section.

n/a	Confirmed	
☐	☒	The exact sample size (*n*) for each experimental group/condition, given as a discrete number and unit of measurement
☐	☒	A statement on whether measurements were taken from distinct samples or whether the same sample was measured repeatedly
☐	☒	The statistical test(s) used AND whether they are one- or two-sided<br>*Only common tests should be described solely by name; describe more complex techniques in the Methods section.*
☒	☐	A description of all covariates tested
☐	☒	A description of any assumptions or corrections, such as tests of normality and adjustment for multiple comparisons
☐	☒	A full description of the statistical parameters including central tendency (e.g. means) or other basic estimates (e.g. regression coefficient) AND variation (e.g. standard deviation) or associated estimates of uncertainty (e.g. confidence intervals)
☐	☒	For null hypothesis testing, the test statistic (e.g. *F*, *t*, *r*) with confidence intervals, effect sizes, degrees of freedom and *P* value noted<br>*Give P values as exact values whenever suitable.*
☒	☐	For Bayesian analysis, information on the choice of priors and Markov chain Monte Carlo settings
☒	☐	For hierarchical and complex designs, identification of the appropriate level for tests and full reporting of outcomes
☒	☐	Estimates of effect sizes (e.g. Cohen's *d*, Pearson's *r*), indicating how they were calculated

Our web collection on statistics for biologists contains articles on many of the points above.

Software and code

Policy information about availability of computer code

Data collection	For flow cytometry analysis and sorting, data was collected using BD FacsDIVA v8.0.1 software (BD FACSAria Fusion). Cancer and immune cell populations were analyzed and sorted based on the markers and gating strategies described in Supplementary Fig. 2.<br>For qRT-PCR, data was collected using an Applied QuantStudio5 machine.<br>For immunofluorescence experiments, data was collected using LAS AF v2.7.3.9723 software (Leica TCS SP5 confocal microscope) and ZEN 3.6 software (ZEISS LSM 980 with Airyscan 2).<br>For immunohistochemistry experiments, data was collected using NIS-Elements BR software (Nikon Eclipse 80i) and ZEN 3.6 software (ZEISS Axioscan 7 Scanner).
Data analysis	Flow cytometry data was analyzed with FlowJo v10.4.2 software.<br>qRT-PCR data was analyzed using SDS 2.3 software.<br>Fiji/ImageJ v1.54d and ZEN Blue 3.6 software were used for all immunofluorescence/immunohistochemistry image analyses.<br><br>GraphPad Prism v8.0.1 and R software v4.0.5 were used for data entry, graph construction and statistical analysis.<br><br>The R package ThresholdROC was used to calculate the best cut-off point for each biomarker, according to Youden's index maximization criterion. Cox proportional hazards models were used to study the association between the percentage of each biomarker and the outcome time to progression/relapse. Results are reported as the hazard ratio (HR) ± 95% confidence interval (CI), and illustrated with a forest plot. The proportionality of risks in the Cox models was verified using Schoenfeld residuals.

For manuscripts utilizing custom algorithms or software that are central to the research but not yet described in published literature, software must be made available to editors and reviewers. We strongly encourage code deposition in a community repository (e.g. GitHub). See the Nature Portfolio guidelines for submitting code & software for further information.

Data

Policy information about availability of data

All manuscripts must include a data availability statement. This statement should provide the following information, where applicable:

- Accession codes, unique identifiers, or web links for publicly available datasets
- A description of any restrictions on data availability
- For clinical datasets or third party data, please ensure that the statement adheres to our policy

Data supporting the findigs of this study are available from the corresponding author upon reasonable request. Source data are provided with this paper.

Research involving human participants, their data, or biological material

Policy information about studies with human participants or human data. See also policy information about sex, gender (identity/presentation), and sexual orientation and race, ethnicity and racism.

Reporting on sex and gender	The findings in our study do not apply to only one gender. Gender was not considered in the study design.
Reporting on race, ethnicity, or other socially relevant groupings	Race/ethnicity was not considered in the study design.
Population characteristics	Details on patient samples can be found in Supplementary Table 1, 2 and 3.<br><br>Cohort 1: paraffin-embedded tissue samples from 14 patient cSCCs with different histopathological grades (G2-G4 grade).<br><br>Cohort 2: paraffin-embedded pre-treatment samples from 14 unresectable locally advanced and metastatic cSCC patients treated with anti-PD-1/PD-L1 therapy (9 patients were males and 5 females, aged between 50 and 88 years). This cohort was divided into two groups according to whether or not cSCC patients showed tumor response to anti-PD-1/PD-L1 therapy. We considered patients as responders if they achieved complete (CR) or partial response (PR) as best response for more than 3 months (n= 7), while non-responder patients were those who had stable (SD) or progressive disease (PD) as their best response (n = 7), according to RECIST v1.1 (see Supplementary Table 1 for more clinical information).<br><br>Cohort 3: paraffin-embedded pre-treatment samples from 19 stage III/IV HNSCC patients treated with anti-PD-1/PD-L1 therapy (16 patients were males and 3 females, aged between 25 and 83 years). This cohort was divided into two groups according to whether or not HNSCC patients showed tumor response to anti-PD-1/PD-L1 therapy. We considered patients as responders if they achieved complete (CR) or partial response (PR) as best response for more than 3 months (n= 6), while non-responder patients were those who had progressive disease (PD) as their best response (n = 13), according to RECIST v1.1 (see Supplementary Table 2 for more clinical information).<br><br>Cohort 4: paraffin-embedded pre-treatment samples from 10 stage IIIC melanoma patients treated with adjuvant anti-PD-1 therapy after resection (8 patients were males and 2 females, aged between 43 and 81 years). In this cohort, we considered patients as non-relapsed if they did not relapse within 18 months of starting adjuvant anti-PD-1 treatment (n = 5), while relapsed patients were those who relapsed within that period (n = 5) (see Supplementary Table 3 for more clinical information).
Recruitment	Our retrospective study includes unresectable locally advanced and metastatic cSCC patients, stage III/IV HNSCC patients and stage IIIC melanoma patients treated with anti-PD-1/PD-L1 therapies between 2016 and 2023 at the Catalan Institute of Oncology (ICO), L'Hospitalet de Llobregat, Barcelona, Spain. Two melanoma samples were collected at La Paz University Hospital, Madrid, Spain. Recruitment was not done specifically for our study.<br><br>Some patients were treated within standard clinical practice and others within several clinical trials. However, all patients received anti-PD-1/PD-L1 drugs as monotherapy.
Ethics oversight	Most cSCC, HNSCC and melanoma patient samples were supplied by the Pathology Unit and Biobank of the Bellvitge University Hospital (Barcelona, Spain). Two melanoma patient samples included in this study were provided by the IdiPAZ Biobank (PT20-0004), integrated in the Biobanks and Biomodels ISCIII Platform.<br><br>This study had approval from the Research Ethics Committee of the Bellvitge University Hospital (Barcelona, Spain) and conformed to the principles of the Declaration of Helsinki (PR392/20 for cSCC, PR381/19 and PR254/21 for HNSCC, and PR186/22 for melanoma). The treatment of the personal data was adjusted to the provisions of the European Data Protection Regulation. All patients were fully informed and provided written informed consent.

Note that full information on the approval of the study protocol must also be provided in the manuscript.

Field-specific reporting

Please select the one below that is the best fit for your research. If you are not sure, read the appropriate sections before making your selection.

☒ Life sciences ☐ Behavioural & social sciences ☐ Ecological, evolutionary & environmental sciences

Life sciences study design

All studies must disclose on these points even when the disclosure is negative.

Sample size	No specific statistical method was used to predetermine the sample size of any experiment. Instead, the sample size was determined based on preliminary experiments to ensure statistical significance between groups and to comply with the principles of the 3Rs in the case of the animal experimentation. The minimun sample size was 3 in all the cases. Sample sizes are indicated in the figure legends.
Data exclusions	No data were excluded from the study.
Replication	For all experiments, biological replicates were used to ensure reproducibility, with an n of at least 3. On the graphs, individual dots represent individual samples used and n is described in figure legends.
Randomization	Mouse experiments: Tumor volume (V (mm3) = π/6 x L x W2; L: largest tumor diameter, W: perpendicular measurement) was monitored by caliper measurements and, when tumors reached a volume of 65 mm3 (5 x 5 mm), mice were randomly assigned over treatment groups. For all the other experiments, including in vitro experiments, randomization was not relevant.
Blinding	Investigators were not blinded to mouse treatment groups. Mice were assigned into therapy groups based on tumor volume and therapy administration was then done based on eartag numbers. After multiple doses of therapy administration, numbers and linked therapies are known to the investigators, making complete blinding difficult. However, experiments were performed by independent investigators. In addition, tumors were weighed upon excision, providing an additional, unbiased read-out.<br><br>For in vitro and immunofluorescence/immunohistochemistry experiments, investigators were not blinded to allocation.

Reporting for specific materials, systems and methods

We require information from authors about some types of materials, experimental systems and methods used in many studies. Here, indicate whether each material, system or method listed is relevant to your study. If you are not sure if a list item applies to your research, read the appropriate section before selecting a response.

Materials & experimental systems

n/a	Involved in the study	
☐	☒	Antibodies
☐	☒	Eukaryotic cell lines
☒	☐	Palaeontology and archaeology
☐	☒	Animals and other organisms
☒	☐	Clinical data
☒	☐	Dual use research of concern
☒	☐	Plants

Methods

n/a	Involved in the study	
☒	☐	ChIP-seq
☐	☒	Flow cytometry
☒	☐	MRI-based neuroimaging

Antibodies

Antibodies used

Mouse flow cytometry:

APC anti-mouse/human CD11b (Clone M1/70, Biolegend, Cat: 101211, 1:250)
PE anti-mouse/human CD11b (Clone M1/70, BD Bioscience, Cat: 557397, 1:250)
PE/Cy7 anti-mouse/human CD11b (Clone M1/70, Biolegend, Cat: 101215, 1:250)
PE/Cy7 anti-mouse CD152 (CTLA-4) (Clone UC10-4B9, Biolegend, Cat: 106313, 1:250)
PE/Cy7 anti-mouse CD155 (PVR) (Clone TX56, Biolegend, Cat: 131511, 1:200)
APC anti-mouse CD206 (MMR) (Clone MR6F3, eBioscience, Cat: 17-2061-80, 1:200)
PE/Cy7 anti-mouse CD223 (LAG-3) (Clone C9B7W, Biolegend, Cat: 125225, 1:250)
PE/Cy7 anti-mouse CD226 (DNAM-1) (Clone 10E5, Biolegend, Cat: 128811, 1:250)
PE/Cy7 anti-mouse CD25 (Clone PC61, Biolegend, Cat: 102015, 1:200)
PE/Cy7 anti-mouse CD274 (PD-L1) (Clone 10F.9G2, Biolegend, Cat: 124313, 1:200)
APC/Cy7 anti-mouse CD279 (PD-1) (Clone 29F.1A12, Biolegend, Cat: 135223, 1:250)
PE/Cy7 anti-mouse CD28 (Clone 37.51, Biolegend, Cat: 102125, 1:250)
APC anti-mouse CD3ε (Clone 145-2C11, Biolegend, Cat: 100311, 1:200)
Anti-mouse CD31 (Clone MEC 13.3, BD Bioscience, Cat: 550274, 1:100)
APC-eF780 anti-mouse CD326 (EpCAM) (Clone G8.8, eBioscience, Cat: 47-5791-82, 1:400)
PE/Cy7 anti-mouse CD366 (TIM-3) (Clone B8.2C12, Biolegend, Cat: 134009, 1:250)
PE/Cy7 anti-mouse CD4 (Clone RM4-5, Biolegend, Cat: 100528, 1:200)
PE anti-mouse CD45 (Clone 30-F11, TONBO, Cat: 50-0451, 1:350)
FITC anti-mouse/human CD49f (a6 integrin) (Clone GoH3, Biolegend, Cat: 313605, 1:10)

PE/Cy7 anti-mouse CD69 (Clone H1.2F3, Biolegend, Cat: 104511, 1:200)
PE anti-mouse CD8a (Clone 53-6.7, Biolegend, Cat: 100707, 1:200)
PE/Cy7 anti-mouse CD80 (Clone 16-10A1, Biolegend, Cat: 104733, 1:250)
APC/Cy7 anti-mouse F4/80 (Clone BM8, Biolegend, Cat: 123118, 1:200)
PE/Cy7 anti-mouse Galectin-9 (Clone 108A2, Biolegend, Cat: 137913, 1:250)
PE/Cy7 anti-mouse Gr-1 (Clone RB6-8C5, Biolegend, Cat: 108415, 1:250)
PE/Cy7 anti-mouse Granzyme B (Clone NGZB, eBioscience, Cat: 25-8898-80, 1:200)
PE/Cy7 anti-mouse IFN-γ (Clone XMG1.2, Biolegend, Cat: 505825, 1:200)
PE/Cy7 anti-mouse Ly-6C (Clone HK1.4, Biolegend, Cat: 128017, 1:250)
APC anti-mouse Ly-6G (Clone 1A8, Biolegend, Cat: 127613, 1:250)
APC anti-mouse Nectin-2/CD112 (Clone 829038, R&D Systems, Cat: FAB3869A, 1:200)
PE anti-mouse NK-1.1 (Clone PK136, Biolegend, Cat: 108707, 1:200)
PE/Cy7 anti-mouse TIGIT (Vstm3) (Clone 1G9, Biolegend, Cat: 142107, 1:250)
7-AAD Viability Staining Solution (Biolegend, Cat: 420403)
DAPI (Thermo Scientific, Cat: 62248)

IF/IHC mouse samples:

Anti-mouse/human CD163, rabbit monoclonal (Clone EPR19518, Abcam, Cat: ab182422, 1:50)
Anti-mouse FoxP3, rabbit monoclonal (Clone D6O8R, Cell Signaling, Cat: 12653, 1:50)
Anti-mouse CD8α, rabbit monoclonal (Clone D4W2Z, Cell Signaling, Cat: 98941, 1:50)
Anti-mouse CD68, rabbit polyclonal (Abcam, Cat: ab125212, 1:200)
Anti-mouse Ly-6G/Ly-6C (Gr-1), rat monoclonal (Clone RB6-8C5, R&D Systems, Cat: MAB1037, 1:200)
DAPI (4',6-diamidino-2-phenylindole, dilactate) (Invitrogen, Cat: D3571, 1:5000)

IF/IHC patient samples:

Anti-mouse/human CD163, rabbit monoclonal (Clone EPR19518, Abcam, Cat: ab182422, 1:50)
Anti-human Granzyme B, rabbit polyclonal (Abcam, Cat: ab4059, 1:100)
Anti-human FoxP3, rabbit monoclonal (Clone D2W8E, Cell Signaling, Cat: 98377, 1:50)
Anti-human PVR/CD155, rabbit monoclonal (Clone D3G7H, Cell Signaling, Cat: 13544, 1:100)
Anti-human CD8α, mouse monoclonal (Clone C8/144B, Abcam, Cat: ab17147, 1:50)
Anti-mouse/human CD80, rabbit polyclonal (Abcam, Cat: ab254579, 1:150)
Anti-human E-cadherin, mouse monoclonal (Clone 36/E-Cadherin, BD Bioscience, Cat: 610182, 1:100)
Anti-human LAG-3, rabbit monoclonal (Clone D2G4O, Cell Signaling, Cat: 15372, 1:100)
Anti-human PD-1, rabbit recombinant (Clone EPR4877(2), Abcam, Cat: ab137132, 1:100)
Anti-human TIM-3, rabbit monoclonal (Clone D5D5R, Cell Signaling, Cat: 45208, 1:100)
Anti-human TIGIT, rabbit monoclonal (Clone E5Y1W, Cell Signaling, Cat: 99567, 1:100)
Anti-human Vimentin, rabbit polyclonal (Abcam, Cat: ab45939, 1:100)
DAPI (4',6-diamidino-2-phenylindole, dilactate) (Invitrogen, Cat: D3571, 1:5000)

Secondary antibodies:

Goat anti-Rat IgG (H+L) Secondary Antibody, Alexa Fluor 546 (Invitrogen, Cat: A-11081)
Donkey anti-Mouse IgG (H+L) Secondary Antibody, Alexa Fluor 568 (Invitrogen, Cat: A10037)
Donkey anti-Rabbit IgG (H+L) Secondary Antibody, Alexa Fluor 568 (Invitrogen, Cat: A10042)
Goat anti-Mouse IgG (H+L) Secondary Antibody, Alexa Fluor 647 (Invitrogen, Cat: A-21235)
Donkey anti-Rabbit IgG (H+L) Secondary Antibody, Alexa Fluor 647 (Invitrogen, Cat: A-31573)
Anti-Rabbit EnVision+ System-HRP Labelled Polymer (Dako, Cat: K4003)

T cell activation:

Anti-CD3e, Functional Grade, hamster monoclonal (Clone 145-2C11, eBioscience, Cat: 16-0031-82, 1ug/ml in suspension)
Anti-CD28, Functional Grade, hamster monoclonal (Clone 37.51, eBioscience, Cat: 16-0281-82, 1ug/ml in suspension)

Mouse treatments:

Mouse IgG2b isotype control (Clone MPC-11, BioXCell, Cat: BE0086, 200ug per dose)
Polyclonal rat IgG isotype control (BioXCell, Cat: BE0094, 200ug per dose)
Anti-CD8a (Clone 2.43, BioXCell, Cat: BE0061, 300ug per dose)
Anti-CTLA-4 (CD152) (Clone UC10-4F10-11, BioXCell, Cat: BE0032, 200ug per dose)
Anti-NK1.1 (Clone PK136, BioXCell, Cat: BE0036, 300 ug per dose)
Anti-PD-L1 (B7-H1) (Clone 10F.9G2, BioXCell, Cat: BE0101, 200ug per dose)
Anti-PD-1 (CD279) (Clone RMP1-14, BioXCell, Cat: BE0146, 200ug per dose)
Anti-TIGIT (Clone 1G9, BioXCell, Cat: BE0274, 200ug per dose)

Validation

All antibodies used are commercially available and were validated by the provider. Relevant information on antibody validation can be found on the manufacturer's websites using the catalog number which is provided for each antibody used and listed in the section above. We used protocols and reccomendations of the manufacturer on validated species.

Eukaryotic cell lines

Policy information about cell lines and Sex and Gender in Research

Cell line source(s)	Primary mouse cSCC cell lines were previously isolated from WD-SCCs, MD/PD-SCCs and PD/S-SCCs generated by orthotopic serial engraftments in male mice, as described in da Silva-Diz, V, Cancer Research, 2016 [8].<br><br>Primary full epithelial cSCC cancer cell lines<br>Primary epithelial EpCAM+ cSCC cancer cell lines<br>Primary mesenchymal EpCAM- cSCC cancer cell lines<br>Primary full mesenchymal cSCC cancer cell lines
Authentication	Cell lines were not authenticated.
Mycoplasma contamination	Cell lines were tested for mycoplasma contamination by PCR and confirmed negative.
Commonly misidentified lines (See ICLAC register)	No commonly misidentified cell lines were used in this study according to ICLAC register.

Animals and other research organisms

Policy information about studies involving animals; ARRIVE guidelines recommended for reporting animal research, and Sex and Gender in Research

Laboratory animals	We have used 6-8-week-old male C57BL6/FVB F1 mice (immunocompetent syngeneic mice). Mice were kept in a pathogen-free facility with a 12-h light/dark cycle at constant temperature (22 ± 2ºC), and with ad libitum access to food and water. Mice were also checked for symptoms of poor health or discomfort during the ICB treatments.
Wild animals	This study did not involve wild animals.
Reporting on sex	Sex was not consider to have a role on the effects observed.
Field-collected samples	This study did not involve field-collected samples.
Ethics oversight	All research involving animals was performed at the Bellvitge Biomedical Research Institute (IDIBELL) animal facility in compliance with the guidelines and protocols approved by the IDIBELL ethics committee and in accordance with Spanish national regulations and to the 3Rs principles (18003, DMAH10402).

Note that full information on the approval of the study protocol must also be provided in the manuscript.

Flow Cytometry

Plots

Confirm that:

- [x] The axis labels state the marker and fluorochrome used (e.g. CD4-FITC).
- [x] The axis scales are clearly visible. Include numbers along axes only for bottom left plot of group (a 'group' is an analysis of identical markers).
- [x] All plots are contour plots with outliers or pseudocolor plots.
- [x] A numerical value for number of cells or percentage (with statistics) is provided.

Methodology

Sample preparation	For flow cytometry analysis and sorting, excised mouse cSCCs were mechanically minced and incubated in RPMI medium with 10% FBS (Life Technologies, 10270106), 20 mM HEPES (Sigma, H3537), 1% Ab/Am (Biowest, L0010-100), 1600 U/ml collagenase type I (Sigma, C0130) and 70 U/ml dispase (Life Technologies, 17105-041), overnight at 37ºC. Cell suspensions were filtered and then depleted of red blood cells by incubating with ACK lysis buffer (Lonza, BP10-548E) for 10 min at room temperature. For endothelial cell depletion, cell suspensions were incubated with a rat anti-mouse CD31 antibody (1:100, BD Bioscience, 550274) for 30 min at 4ºC, and then with Dynabeads anti-rat IgG (1:33, Life Technologies, 11035) for 30 min at 4ºC. For cell-surface staining, cells were blocked with 1 mg/ml IgG (Sigma, I5381) and stained with a cocktail of cell-surface antibodies in staining buffer (5% FBS in PBS) for 30 min at 4ºC (see Supplementary Table 4). Cells were then washed with 0.5% BSA, 2 mM EDTA in PBS, and resuspended in analysis buffer (2% FBS, 2 mM EDTA in PBS). Viability was assessed with DAPI (Thermo Scientific, 62248).<br><br>For intracellular cell staining, cells were stimulated with Leukocyte Activation Cocktail with GolgiPlugTM (BD Bioscience, 550583) for 4 h at 37ºC, stained using the LIVE/DEADTM Fixable Dead Cell Stain Kit (Life Technologies, L34963) for 30 min at 4ºC, and incubated with a cocktail of cell-surface antibodies for 30 min at 4ºC (see Supplementary Table 4). Cells were then fixed with PFA 4% (Electron Microscopy Sciences, 15710-S) for 20 min at 4ºC, permeabilized with Permeabilization Buffer 1X (Life Technologies, 00-8333-56) for 15 min at 4ºC, and stained with antibodies recognizing intracellular antigens for 30 min at

4ºC (see Supplementary Table 4). Cells were then washed with Permeabilization Buffer 1X, and resuspended in analysis buffer (1% PFA in PBS).

CD8+ T cells were isolated from the spleens of C57BL6/FVB F1 mice bearing epithelial or mesenchymal cSCCs. Spleens were mashed in PBS with 2% FBS through a 70-µm filter. Red blood cells were lysed with ACK lysis buffer for 5 min on ice. CD8+ T cells were isolated using the MojoSortTM Mouse CD8 T Cell Isolation Kit (Biolegend, 480008), and then were labeled with 2.5 µM CellTraceTM Violet dye (Thermo Fisher, C34557) for 20 min at 37ºC. Purified CD8+ T cells were resuspended in T-cell medium (RPMI with 10% FBS, 1% non-essential amino acids, 1% Na-pyruvate, 1% L-glutamine, 1% P/S, 50 µM β-mercaptoethanol) and activated in vitro by incubating with 1 µg/ml of anti-CD3e (clone 145-2C11, eBioscience, 16-0031-82) and 1 µg/ml of anti-CD28 (clone 37.51, eBioscience, 16-0281-82) antibodies. Epithelial and mesenchymal cSCC cancer cells were added to CD8+ T cells at 1:1 ratio on day 2 after T cell activation. After 2 days of co-culture, CD8+ T cells were collected, stained with a cocktail of antibodies in staining buffer (5% FBS in PBS) for 30 min at 4ºC, washed with 0.5% BSA, 2 mM EDTA in PBS, and resuspended in analysis buffer (2% FBS, 2 mM EDTA in PBS). Viability was assessed with 7-AAD Viability Staining Solution (Biolegend, 420403) or LIVE/DEADTM Fixable Dead Cell Stain Kit (1:1000, Life Technologies, L34963).

Instrument

Flow cytometry sorting and analysis were performed on a BD FACSAria Fusion equipment.

Software

BD FacsDIVA v8.0.1 software was used for data collection.
Data analysis including quantification and data visualization were performed using FlowJo v10.4.2 software.

Cell population abundance

Cells were gated according to well defined marker combinations.

Gating strategy

All gating strategies are described in Supplementary Fig. 2.

For FACS comparison between the levels of different markers among groups, cells were gated as follows:
1- FSC-A/SSC-A to identify cells of interest.
2- FSC-A/FSC-W to exclude doublets/complexes.
3- DAPI, 7-AAD or LIVE/DEAD Fixable Violet to identify live/dead cells (we use these 3 markers due to some incompatibilities with other fluorochromes).
4- Comparison of expression levels. The cut-off of the negative population was established according to the unstained samples.
5- Cancer cells: GFP+CD45-
5.1- Epithelial cancer cells: GFP+CD45-EpCAM+
5.2- Mesenchymal cancer cells: GFP+CD45-EpCAM-
6- CD45+ for general immune infiltration
6.1- T lymphocytes: CD45+CD3+CD11b-
6.2- CD8 T cytotoxic: CD45+CD3+CD8+CD4-
6.3- NK cells: CD45+CD3-NK-1.1+
6.4- Myeloid cells: CD45+CD11b+CD3-
6.5- PMN-MDSCs: CD11b+ Ly6Clo Ly6G+
6.6- M-MDSCs: CD11b+ Ly6Chi Ly6G-
6.7- Macrophages: CD45+ CD11b+ F4/80+
6.8- M1-like macrophages: CD11b+ F4/80+ CD206-
6.9- M2-like macrophages: CD11b+ F4/80+ CD206+

☒ Tick this box to confirm that a figure exemplifying the gating strategy is provided in the Supplementary Information.
